# Crystal structure of the Rab33B/Atg16L1 effector complex

**DOI:** 10.1038/s41598-020-69637-0

**Published:** 2020-07-31

**Authors:** Janina Metje-Sprink, Johannes Groffmann, Piotr Neumann, Brigitte Barg-Kues, Ralf Ficner, Karin Kühnel, Amanda M. Schalk, Beyenech Binotti

**Affiliations:** 1 Department of Neurobiology, Max-Planck-Institute for Biophysical Chemistry, 37077 Göttingen, Germany; 2 Department of Molecular Structural Biology, Institute of Microbiology and Genetics, GZMB, Georg-August-University Göttingen, 37077 Göttingen, Germany; 3 Present Address: Institute for Biosafety in Plant Biotechnology, Julius Kuehn-Institute, 06484 Quedlinburg, Germany; 4 Present Address: Nature Communications, 4 Crinan Street, London, N1 9XW UK; 5 Present Address: Department of Biochemistry and Molecular Genetics, University of Illinois at Chicago, Chicago, IL 60607 USA; 6 Present Address: Department of Biochemistry, University of Würzburg, 97074 Würzburg, Germany

**Keywords:** X-ray crystallography, GTP-binding protein regulators

## Abstract

The Atg12-Atg5/Atg16L1 complex is recruited by WIPI2b to the site of autophagosome formation. Atg16L1 is an effector of the Golgi resident GTPase Rab33B. Here we identified a minimal stable complex of murine Rab33B(30–202) Q92L and Atg16L1(153–210). Atg16L1(153–210) comprises the C-terminal part of the Atg16L1 coiled-coil domain. We have determined the crystal structure of the Rab33B Q92L/Atg16L1(153–210) effector complex at 3.47 Å resolution. This structure reveals that two Rab33B molecules bind to the diverging α-helices of the dimeric Atg16L1 coiled-coil domain. We mutated Atg16L1 and Rab33B interface residues and found that they disrupt complex formation in pull-down assays and cellular co-localization studies. The Rab33B binding site of Atg16L1 comprises 20 residues and immediately precedes the WIPI2b binding site. Rab33B mutations that abolish Atg16L binding also abrogate Rab33B association with the Golgi stacks. Atg16L1 mutants that are defective in Rab33B binding still co-localize with WIPI2b in vivo. The close proximity of the Rab33B and WIPI2b binding sites might facilitate the recruitment of Rab33B containing vesicles to provide a source of lipids during autophagosome biogenesis.

## Introduction

During macroautophagy, denoted here as autophagy, a growing isolation membrane (also called the phagophore) engulfs cytoplasmic content leading to the formation of a double-membraned vesicle, the autophagosome. Autophagosomes then fuse with the vacuole in yeast and plants or lysosomes in animal cells, where their content is degraded ^1–3^. Each step in this pathway is mediated by a set of autophagy-related (Atg) proteins that are recruited in a sequential and hierarchical manner to the forming autophagosome^4^.

Two ubiquitin-like (Ubl) conjugation systems are essential for autophagosome formation^5^. One catalyzes the C-terminal lipidation of the Ubl protein Atg8 with phosphatidylethanolamine^6^. The mammalian Atg8 homologue, the microtubule-associated protein 1 light chain 3 (LC3), is also lipidated^7^. The second Ubl conjugation pathway links the Ubl protein Atg12 covalently to Atg5^8^. The Atg12–Atg5 conjugate further interacts with Atg16^9–11^. Atg16 dimerizes through its coiled-coil domain^12^ and the Atg12–Atg5/Atg16 complex associates with the outer surface of the growing isolation membrane and dissociates from the membrane shortly before or after completion of autophagosome formation^13^. The Atg12-Atg5/Atg16 complex has E3 ligase activity towards Atg8 and LC3 lipidation^14,15^.

Atg16 consists of an N-terminal Atg5 binding region and a coiled-coil domain^9^. Mammalian Atg16L homologues contain an additional C-terminal WD40 domain that is lacking in yeast Atg16^11^. Multiple mammalian Atg16L isoforms exist^11,16^. The structures of the individual Atg16 domains have been determined, including the complexes of the N-terminal regions of yeast and human Atg16 bound to Atg5 and the Atg12–Atg5 conjugates, respectively^17–20^. The structures of the yeast Atg16 dimeric coiled-coil domain^21^ and the WD40 domain of human Atg16L1^22^ are also known.

Atg16L1 binds mammalian WIPI2b (WD-repeat PtIns3P effector protein 2b) and this interaction is important for the membrane recruitment of Atg12-Atg5/Atg16L1 during the early stages of starvation induced autophagy^23^. The WIPI2b binding site is localized at the C-terminal end of the Atg16L1 coiled-coil domain and comprises residues 208–241^23^. WIPI1-4 are the mammalian homologues^24^ of the PROPPIN family (β-propellers that bind polyphosphoinositides)^25^. PROPPINs play a role in autophagy and are seven-bladed β-propellers with two lipid binding sites that are specific for PtdIn3P and PtdIns(3,5)P_2_^26–28^. The yeast PROPPIN Atg21 interacts with both the Atg16 coiled-coil domain and Atg8 and determines the site of Atg8 lipidation at the pre-autophagosomal structure (PAS) from which autophagosome biogenesis originates^29^.

Besides WIPI2b, the Atg16L1 coiled-coil domain also interacts with the Golgi resident Rab33B^30^. Rab proteins are small GTPases that cycle between an inactive GDP bound form and an activated GTP bound state, where they interact with specific effector molecules. Rab proteins are key regulators of intracellular membrane trafficking processes and are also involved in the formation and maturation of autophagosomes and their fusion with lysosomes^31–33^.

Rab33B interacts with Atg16L1 in a GTP dependent manner and therefore Atg16L1 is an effector of Rab33B. The Rab33B binding site was mapped to the C-terminal part of the Atg16L1 coiled-coil domain comprising residues 141–265^30^. The interaction between Atg16L1 and Rab33B might facilitate the recruitment of Golgi derived vesicles to the growing isolation membrane. The Rab33B structure is known^34^. Another link between Rab33B and autophagy comes from the observation that its GTPase-activating protein (GAP) OATL1 binds Atg8 and that both Rab33B and OATL1 are involved in the fusion step between autophagosomes and the lysosome^35^.

Here, we have identified a minimal Atg16L1 construct that binds Rab33B and determined the crystal structure of the Rab33B-Atg16L1 (153–210) effector complex at 3.47 Å resolution. Based on the structure, we designed Rab33B and Atg16L1 point mutants that disrupt Rab33B–Atg16L1 complex formation, analyzed them in pull-down assays and characterized their cellular localization. This experiments have led to the conclusion, that the Rab33B binding site immediately precedes the WIPI2b binding site and mutations within the Atg16L1-binding region showed a marked or even complete loss of Golgi-localization. We also observed that Atg16L1 mutants deficient in Rab33B binding still localize with WIPI2b in vivo.

## Results

### Identification of the minimal Rab33B binding site in Atg16L1

**Figure 1:**
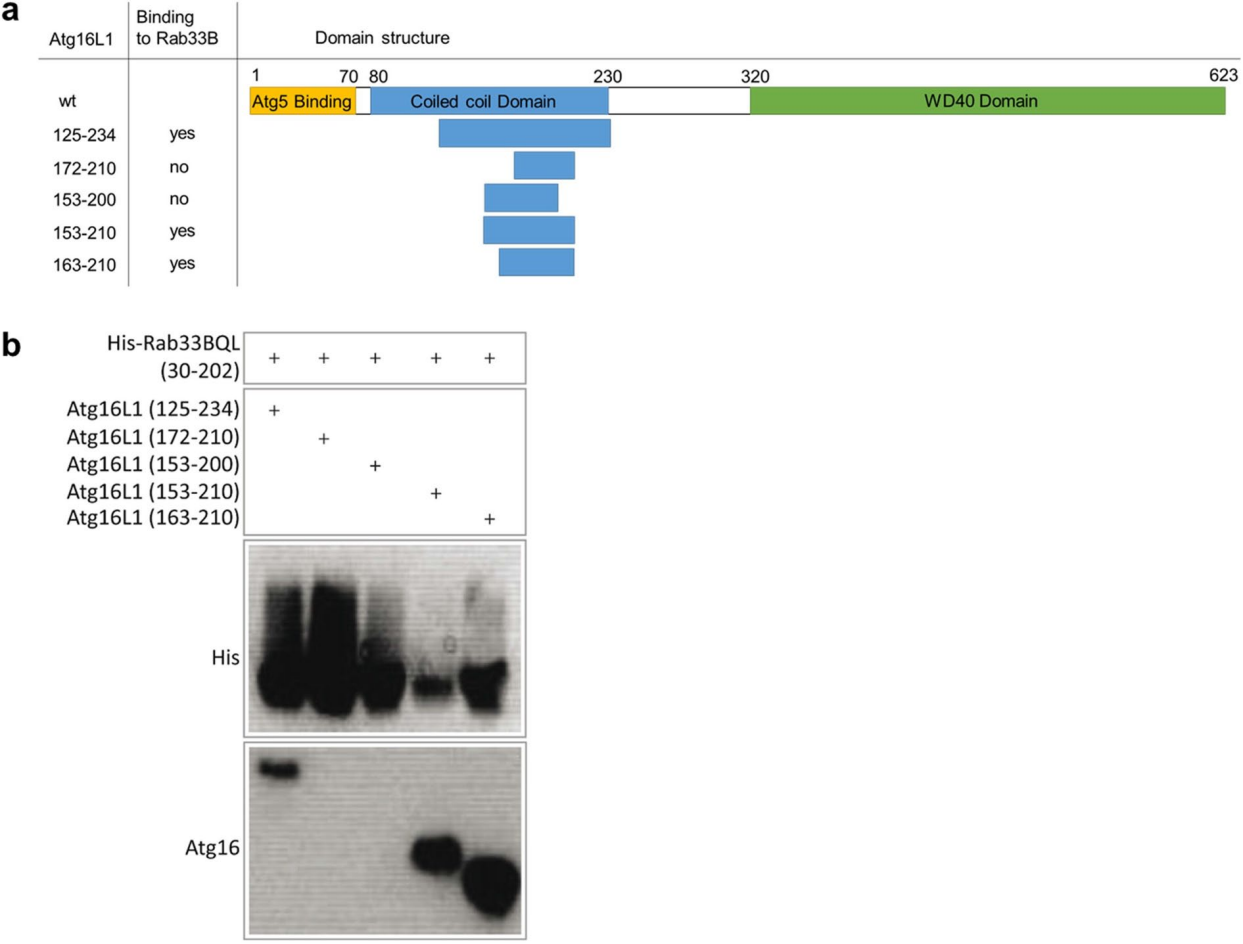
Identification of the minimal Rab33B binding site in Atg16L1. **(a)** Domain structure of full-length Atg16L1 and the truncated mAtg16L1 constructs prepared for Rab33B (30–202)Q92L binding. Yes or no indicates whether complex formation was observed in the pull down experiments. (**b)** Ni-Sepharose beads pull down experiments of His-Rab33B(30–202)Q92L co-expressed with the truncated Atg16L1 constructs. Samples were run on Schägger gels after elution from the Ni-Sepharose beads and then blotted onto nitrocellulose membranes. Membranes were probed with a rabbit anti-Atg16L primary antibody and HRP-labeled goat anti-rabbit IgG secondary antibody. Uncropped blots are shown in Figure S6.

Itoh et al.^30^ mapped the Rab33B binding site to residues 141–265 in Atg16L1, which comprises the C-terminal part of the coiled-coil domain and the region connecting it with the WD40 domain. Our goal was to identify an Atg16L1 construct that comprises the minimal Rab33B binding site for crystallization of the complex. We used the previously crystallized murine Rab33B(30–202) construct^34^ for co-expression experiments. Since Atg16L1 is an effector of Rab33B, the GTPase deficient Rab33B(30–202) Q92L mutant was used to stabilize the Rab33B/Atg16L1 complexes. His-tagged murine Rab33B(30–202) Q92L and different untagged murine Atg16L1 constructs (Fig. 1a) were co-expressed from the pETDuet-1 plasmid (Merck) in *E. coli* and purified with Ni-Sepharose beads. Atg16 binding was detected by Western Blotting (Fig. 1b). Besides the Atg16L1(125–234) construct that spans the entire predicted Rab33B binding site, Atg16L1(153–210) and Atg16L1(163–210) also formed complexes with Rab33B(30–202) Q92L. Further truncations of Atg16L1 to residue 172 or 200 at the C-terminal end abolished complex formation. Rab33B Q92L-Atg16L1(153–210) and Rab33B Q92L-Atg16L1(163–210) purifications were scaled up. The Rab33B Q92L-Atg16L1(153–210) complex was more stable during purification and yielded small needle crystals, whereas the Rab33B Q92L-Atg16L1(163–210) complex did not crystallize.

### The Rab33B–Atg16L1 crystal structure

**Figure 2:**
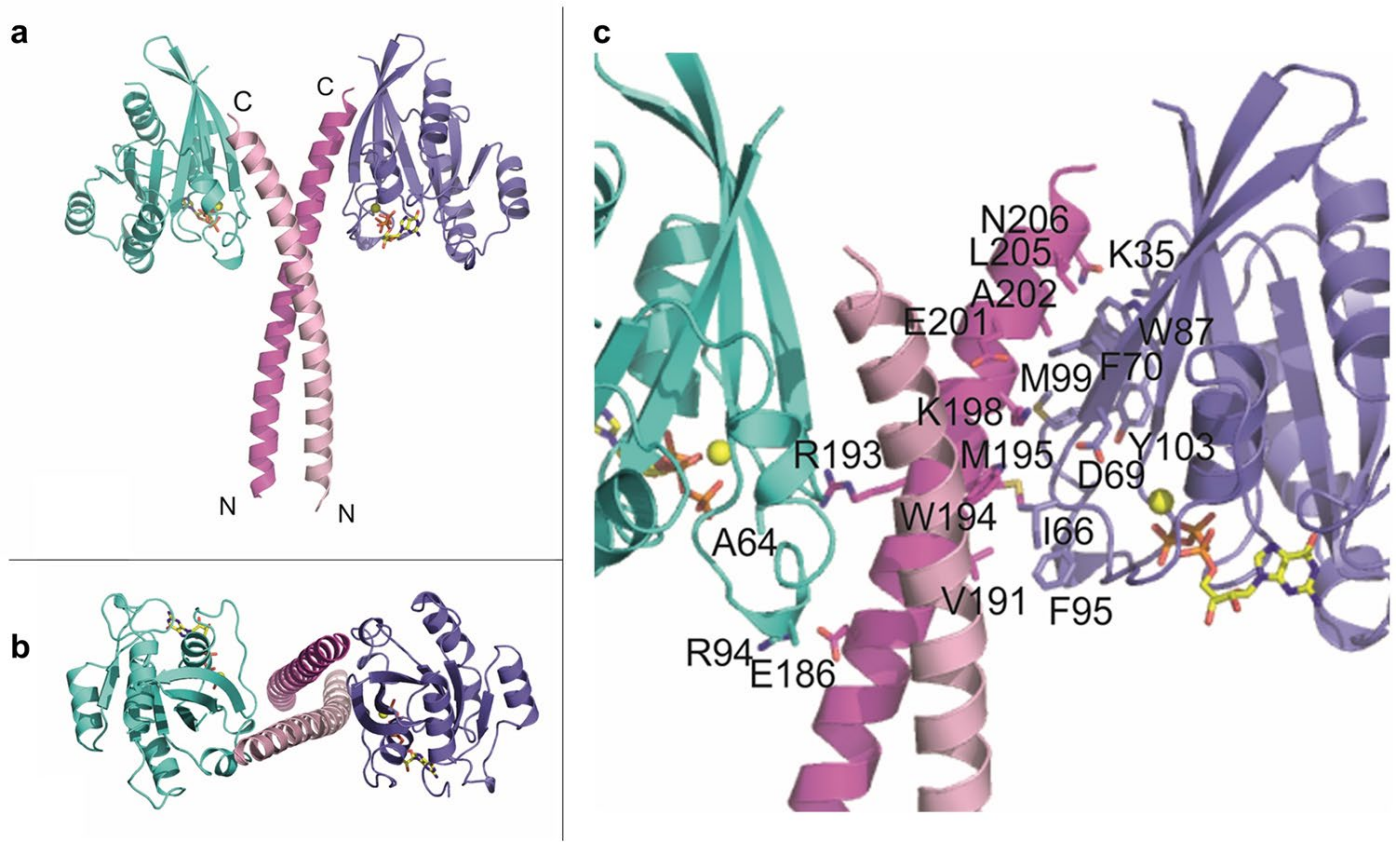
Structure of the Rab33B/Atg16L1(153–210) complex. **(a)** Overall structure of the complex. Two Rab33B molecules interact with one mAtg16L1 dimer. Rab33B molecules are colored turquoise and purple and mAtg16L1 chains are shown in two pink shades. GTP is drawn in stick representation and the Mg^2+^ ions are shown as yellow spheres. (**b)** Top view of the complex. (**c)** Close up showing details of the interactions between Atg16L1 and Rab33B.

The mRab33B–mAtg16L1 (153–210) complex structure was determined at 3.47 Å resolution. The structure was solved by molecular replacement using the structures of murine Rab33 (PDB accession code: 1Z06^34^ and the *S. cerevisiae* Atg16 coiled-coil domain (PDB accession code: 3A7O^12^ as search models. The Rab33B/Atg16 complex is a heterotetramer and the model comprises an Atg16L1 coiled-coil dimer and two Rab33B molecules. The parallel Atg16L1 coiled-coil dimer is located in the center (Fig. 2a,b). The two mRab33B molecules bind to the diverging C-terminal ends of the coiled-coil and are not in direct contact. There are three Rab33B/Atg16L1 complexes in the asymmetric unit, which align with a root-mean-square deviation (RMSD) of 0.60 Å. While the Rab33B molecules are very similar, there are small differences between the N-termini of Atg16L1 dimer (Figure S1).

**Figure 3:**
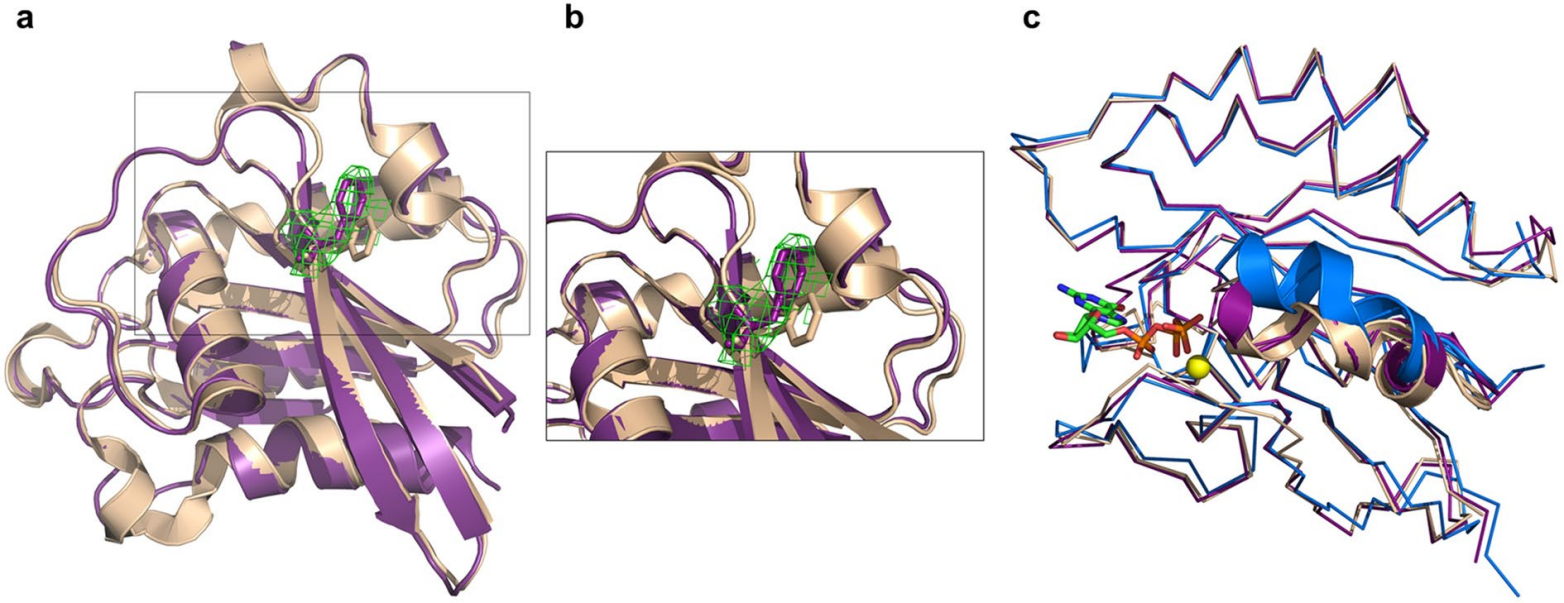
Conformational changes of Rab33B upon Atg16L1 binding.** (a)** Cartoon representation of Atg16L1 bound mRab33B purple superimposed with GppNHp-bound Rab33 (PDB accession code 1Z06^34^ shown in wheat. Residue F70 is shown in stick representation in purple for Rab33B and colored wheat for GppNHp-bound Rab33. (**b)** Close up view of Rab33B residue F70. The omit F_o_–F_c_ difference electron density map (green) is contoured at + 2σ. (**c)** Ribbon representation of mRab33B in purple with bound GTP shown as green stick model and Mg^2+^ as a yellow sphere superimposed with GppNHp-bound Rab33 (PDB accession code 1Z06^34^ in wheat and GDP-bound Rab33 (PDB accession code 2G77^34^ in blue. The Switch II region is shown in cartoon representation.

The N-terminal end of Atg16L1 is positioned in the proximity of the opposing Rab33B molecule, and residues E186 of the Atg16L1 dimer form salt bridges with nearby positioned Rab33B R94 residues localized in switch II, while hydrogen bonds between R193 of the Atg16L1 dimer and the carbonyl oxygens of Rab33B A64 in switch I are formed (Fig. 2c and S2). The diverging Atg16L1 helices then interact with switch I, switch II and the interswitch regions of the nearby Rab33B molecule through hydrophobic interactions and a salt bridge between Atg16L1 K198 and Rab33B D69 (Fig. 2c and S2). At the C-terminal end, the Rab33B aromatic triad, composed of residues F70, W87 and Y103, forms hydrophobic interactions with Atg16L1. Atg16L1 N206 forms a hydrogen bond with Rab33B K35. The structures of Atg16L1 bound Rab33B and free GppNHp-bound Rab33B ^34^ are very similar (Fig. 3c) with the exception of the F70 side chain, which adopts a different conformation to accommodate Atg16L1 binding (Fig. 3a,b). Upon GTP binding the conformation of the switch II region changes to the GDP-bound Rab33B^34^.

**Figure 4:**
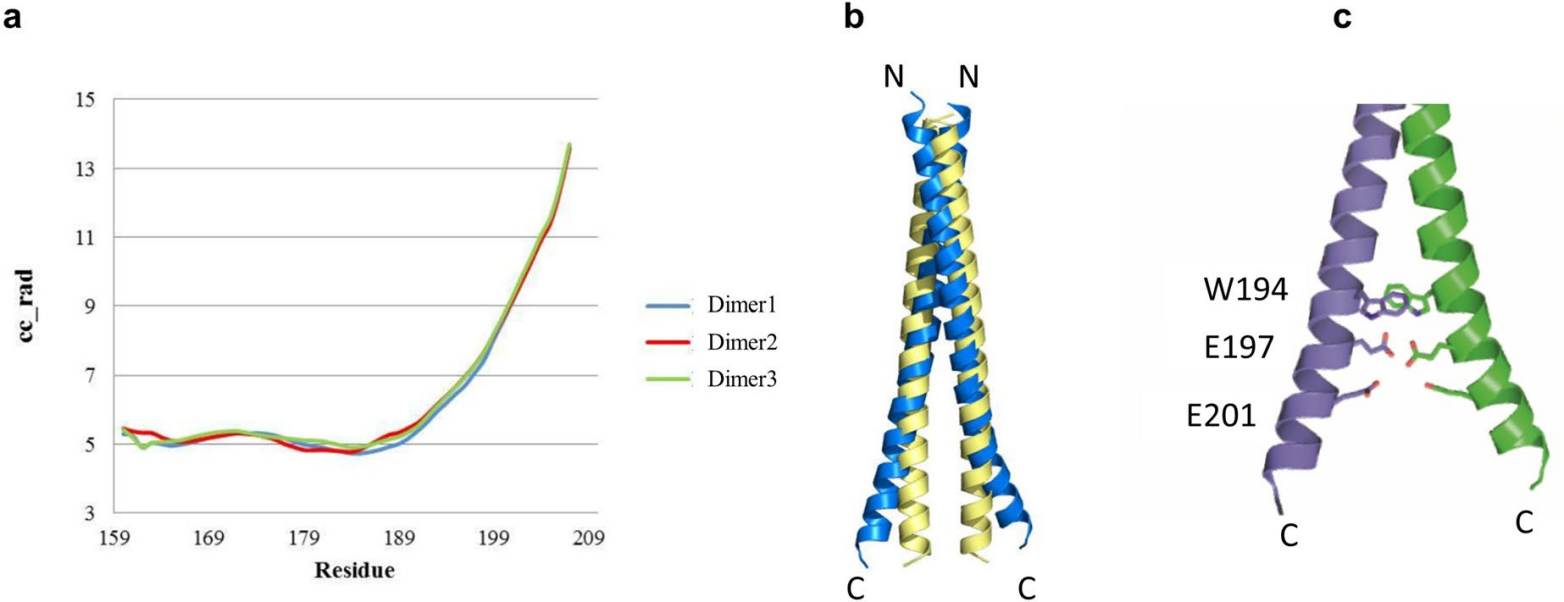
Analysis of the Atg16L1 coiled coil domain. **(a)** Twister^36^ analysis of the Atg16L1 coiled coil geometric parameters for the three Atg16L1 dimers in the asymmetric unit. The local coiled-coil radius in Å (cc_rad) is shown as a function of residue number. Divergence of the α-helices starts at residue 189. (**b)** Cartoon representation of Atg16L1 coiled coil dimer colored blue superimposed with an ideal Atg16L1 coiled coil dimer model shown in yellow. The ideal Atg16L1 coiled coil dimer model was made with CCBuilder Version 1.0^37^. (**c)** The divergence of the α-helices can be explained with steric constraints due to the bulky side chains of W194 and repulsive forces between residues E197 and E201.

The geometric parameters of the Atg16L1 coiled-coil domain were analyzed with the program TWISTER^36^^,^ which calculates the local coiled-coil radius along the coiled-coil axis as a function of residue number (Fig. 4a). The two helices diverge from residue 189 onwards due to the bulky side chain of W194 at the coiled-coil core and repulsive forces between residues E197 and E201 of the two α-helices (Fig. 4c). The Atg16L1 crystal structure was superimposed with an ideal Atg16L1 coiled-coil dimer that was generated with CCBuilder^37^ to visualize the diverging helices (Fig. 4b).

The crystal structure explains the results of the co-expression experiments, where a truncation of the Atg16L1 C-terminus from 210 to 200 abolished Rab33B binding because part of the interaction region is then lost. The Rab33B complex with the Atg16L1 construct (153–210) is likely more stable than the Rab33B/Atg16(163–210) complex because the longer N-terminus stabilizes the coiled-coil domain.

### Analysis of Rab33B/Atg16L1 mutant complex formation

We mutated Rab33B and Atg16L1 residues with the aim of disrupting Rab33B and Atg16L1 interactions in order to further validate the crystal structure and to probe their effects on complex formation in vivo. Atg16L1 K198 was mutated to an alanine to disrupt its salt bridge with Rab33B D69. Atg16L1 A202 is positioned in proximity to Rab33B residues W87 and M99 and was mutated to a tryptophan. At the C-terminal end of the interaction site, Atg16L1 N206 was changed to a lysine in order to abolish its interaction with Rab33B K35. The Rab33B aromatic triad residues F70 and W87 were mutated to F70A, F70E and W87A.

**Figure 5:**
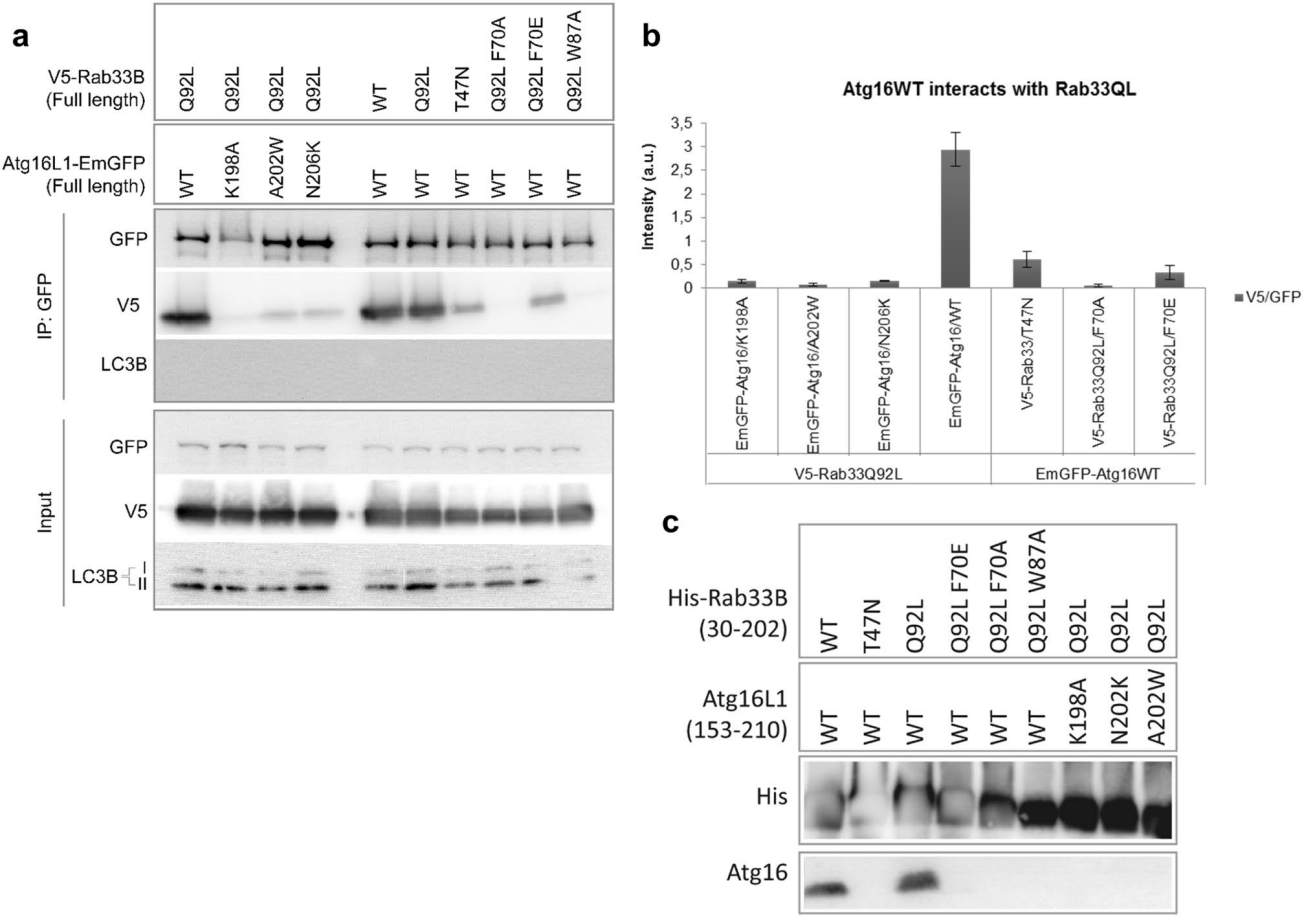
Effects of Rab33B and Atg16 mutations on complex formation analyzed by GFP co-immunoprecipitation and Ni-sepharose pulldown experiments. **(a)** GFP Co-immunoprecipitation. Overexpression was done in HEK293 cells. Western blots were probed with either anti-GFP, anti-V5 or anti-LC3B antibodies. *IP* immunoprecipitation. (**b)** Quantification of the western blot band intensities shown in **(a)**. Band intensities were calculated by normalizing the V5-IP blot band intensities against GFP-IP intensity. Error bars represent standard errors (SE) of three independent biological replicates. (**c)** His-Rab33B(30–202) mutants were co-expressed with Atg16L1(153–210) wild-type and mutants. Samples were run on Schägger gels after elution from the Ni-Sepharose beads and then blotted onto nitrocellulose membranes. Membranes were probed with rabbit anti-Atg16L primary antibody and goat anti-rabbit IgG (HRP labeled) secondary antibody. Uncropped images of blots are shown in Figures S7 and S8.

Untagged Atg16L1(153–210) and His-Rab33B(30–202) were co-expressed and purified with Ni-Sepharose beads. First, the interaction of wild-type Atg16L1 with wild-type Rab33B, the GTPase deficient Q92L mutant and the dominant-negative T47N mutant were analyzed (Fig. 5c). Atg16L1 co-purified with both wild-type and the Rab33B Q92L mutant but did not bind the dominant-negative T47N mutant, which is consistent with earlier observations^30^. The three Rab33B Q92L F70A, F70E and W87A mutants did not interact with wild-type Atg16L1. The single-site Atg16L1 mutants K198A, A202W and N206K were also sufficient to abolish Atg16L1 Rab33B complex formation.

### Co-immunoprecipitation and pull down assays

Next, we analyzed the effects of these mutants on complex formation of the full-length proteins in vivo by expressing C-terminal GFP-tagged Atg16L1 and N-terminally V5 epitope-tagged Rab33B in HEK293T cells and tested complex formation with GFP (Fig. 5a) and V5 (Figure S3a) via co-immunoprecipitation assays. GFP-Atg16L1 WT co-precipitated with both Rab33B WT and Q92L but only slightly with Rab33B T47N (Fig. 5a,b). Complex formation of Rab33B Q92L with the three Atg16L1 K198A, A202W and N206K mutants was disrupted entirely (Fig. 5a,b). The Rab33B W87A and F70A mutations abolished Atg16L1 binding, while Rab33B Q92L F70E binding was strongly reduced in comparison with Rab33B Q92L.

### Cellular localization of the Rab33B and Atg16L1 mutants

**Figure 6:**
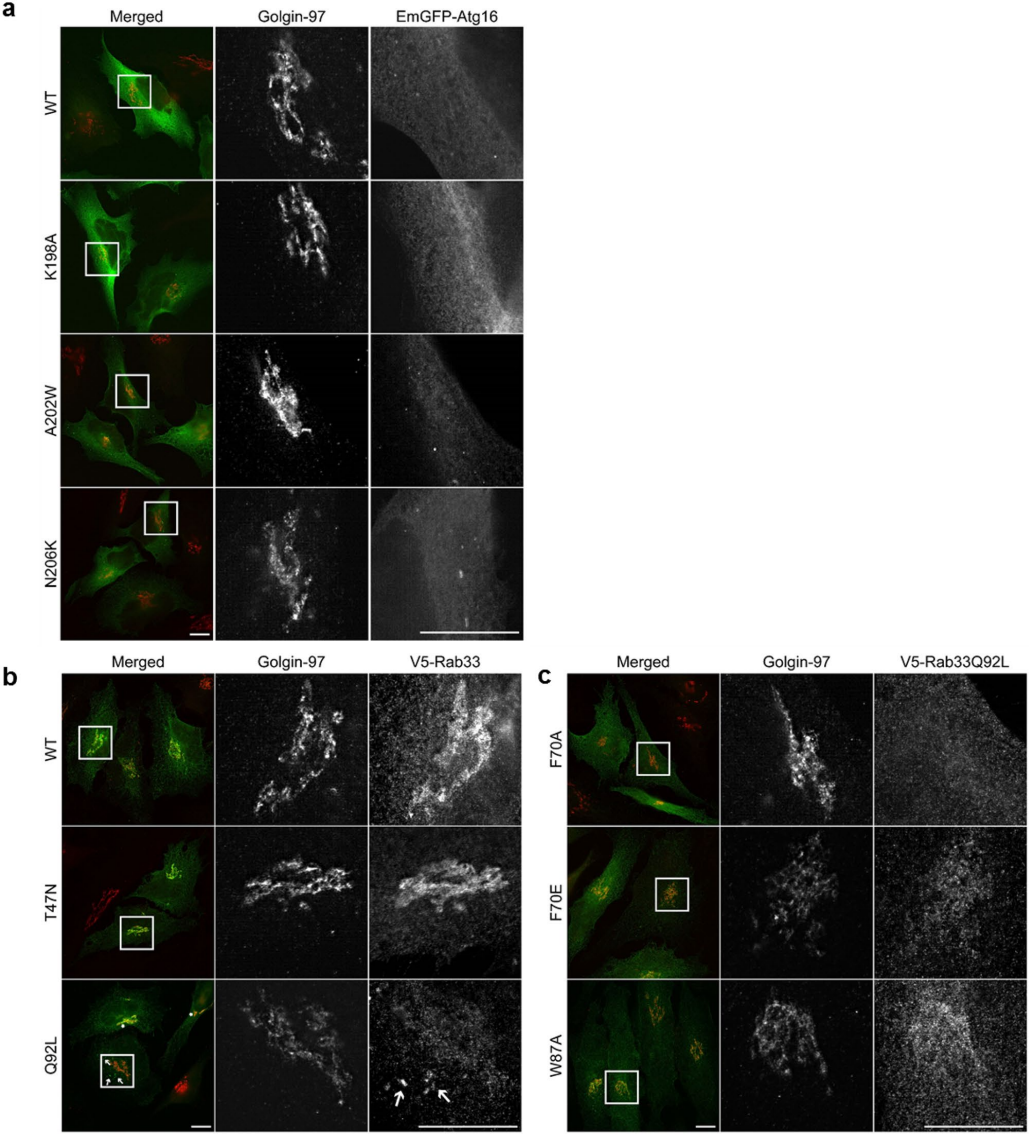
Intracellular distribution of Atg16L1-EmGFP and V5-Rab33B variants in HeLa cells. Overexpression of both Atg16L1-EmGFP and V5-Rab33B variants do not alter Golgi morphology (**a–c**). (**a)** Atg16L1 shows mainly cytosolic distribution with small puncta structures; **b:** V5-Rab33B WT, T47N are mainly found on the Golgi stack (**b** rows 1 and 2) while Q92L (row 3) shows both Golgi (asterisks) and cytoplasmic puncta localization (arrows). (**c)** V5-Rab33Q92L mutants F70A, F70E and W87A lose their Golgi association. Scale bar, 10 µm.

Rab33B is a Golgi resident small GTPase that strongly associates under physiological condition with Golgi stacks^30^. We used fluorescence microscopy to study the intracellular localization of Rab33B and Atg16L1. In order to analyze whether the Atg16L1 or Rab33B mutations disrupt Golgi morphology in cells, we transiently transfected either Atg16L1 variants (Fig. 6a) or Rab33 variants (Fig. 6b,c) in HeLa cells and co-stained with the Golgi marker, Golgin-97. The overexpression of either Atg6L1 or Rab33B variants did not affect Golgi morphology (Fig. 6a–c).

The overexpression of the various Atg16L1 mutants did not significantly change their cellular distribution. In contrast, the expression of the Rab33 Q92L mutants showed a diffused staining and an alteration of Golgi localization from either weak association as observed for Rab33 Q92L F70E or W87A (Fig. 6c, row 2, 3) to complete loss of association when expressing Rab33 Q92L F70A (Fig. 6c, row 1). This implies that these Rab33 interface point mutations are not only essential for its interaction with Atg16L1 but are in addition important for its recruitment to the Golgi.

We then checked the co-expression of Atg16L1 with the different Rab33B variants (Figure S4). We co-transfected Cos cells with V5 epitope-tagged Rab33B and GFP-tagged Atg16L1 variants (Figure S4). As reported by Itoh et al., 2008^30^ co-expression of WT Atg16L1 and either WT Rab33B or Rab33B Q92L showed punctate structures which strongly co-localized (Figure S4a, first and second row). These punctate structures accumulated at the perinuclear region. When WT Atg16L1 was co-expressed with Rab33B Q92L (Figure S4a, second row), larger punctuate structures were observed when compared to the WT Rab33B transfected cells (Figure S4a, first row). In contrast, co-expression of the dominant-negative mutant Rab33B T47N and WT Atg16L1 strongly reduced the number of punctuate structures (Figure S4a, third row) but showed strong association with the Golgi (shown in HeLa cells in Fig. 6b, row 3).

Next, the effect of the complex-disrupting Atg16L1 and Rab33B mutations on cellular localization was analyzed. Co-expression of WT Atg16L1 with the Rab33B Q92L F70A, F70E and W87A point mutations led to an almost complete loss of punctuate structures (Figure S4a, rows 4 to 6). Similarly, co-expression of Rab33B Q92L with the Atg16L1 K198A, A202W or N206K mutants strongly reduced punctate structures formation (Figure S4b). A few punctate Atg16L1-positive structures were observed in some cells, but here Atg16L1 did not localize with Rab33B Q92L. There were no morphological differences between cells where the Rab33B–Atg16L1 complex occurred and cells where Rab33B and Atg16L1 interactions were disrupted. The results of the in vivo co-localization experiments are consistent with the findings from the co-immunoprecipitation experiments with the Rab33B and Atg16L1 mutants which showed that Rab33B mutants are unable to interact with Atg16L1 and vice versa.

### The Atg16L1–Rab33B interface mutants localize with WIPI2B in vivo

**Figure 7:**
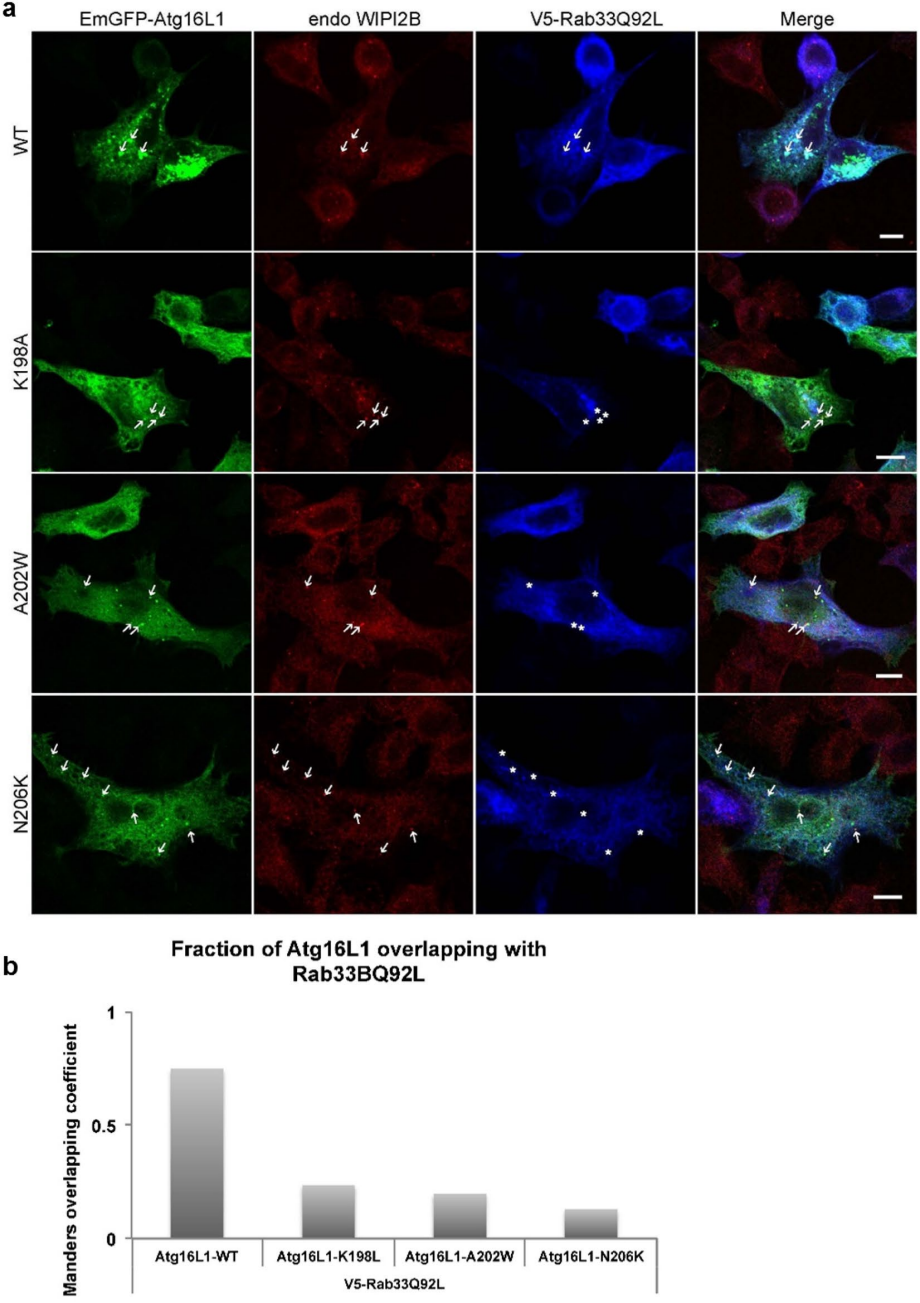
Colocalization of EmGFP-Atg16L1, endo-WIPI2 and V5-Rab33B Q92L.** (a)** Endo-WIPI2b and V5-Rab33B Q92L were transiently expressed with EmGFP-Atg16L1 WT or mutants K198A, A202W or N206K in HeLa Cells. Arrows indicate colocalization, asterisks show no overlap. Scale bar, 10 µm. Figures are representative for three biological replicates. **(b)** Fraction of Atg16L1 overlapping with Rab33B Q92L as measured with Mander´s overlapping coefficient was analyzed using the ImageJ plugin JACoP.

Another recently discovered binding partner of Atg16L1 is WIPI2b, which was shown to be important for the recruitment of Atg16L1 on the preautophagosome membrane^23^. WIPI2b, an orthologue of the yeast core autophagy protein Atg18^24^ is very similar to yeast Atg21. It binds to PtdIn3P at the phagophore, scaffolds the Atg5 ~ Atg12/Atg16L1 complex and brings LC3 close to the membrane and in that manner promotes LC3 lipidation. The WIPI2b binding site of Atg16L1 (207–242) is directly bordering, but is not identical with, the Rab33B binding site (191–208). To further examine the relationship between Rab33B-Atg16L1 and WIPI2B–Atg16L1 complex formation, all three proteins were fluorescently labeled simultaneously (Fig. 7a). While co-localization between Atg16L1 WT, V5-Rab33Q92L and WIPI2b was shown to occur at the level of small punctate structures (Fig. 7a, first row), the Atg16L1 mutants were unable to bind Rab33Q92L (Fig. 7a, row 2 to 4 and 7b), although they were still capable to co-localize with endogenous WIPI2b (Fig. 7a, arrows). These results indicate that under physiological conditions, both WIPI2B and Rab33B can be found simultaneously on the same Atg16L1 compartment.

## Discussion

Atg16L1 was proposed to be an effector of Rab33B, and the Rab33B binding site was previously mapped to the region comprising residues 141–265 that includes part of the coiled-coil domain and the region connecting it with the WD40 domain^30^. Here we identified murine Atg16L1(153–210) as the minimal construct that forms a stable complex with Rab33B Q92L by testing various truncated Atg16 constructs for co-purification with His-tagged Rab33B Q92L. The crystal structure of the Atg16L1(153–210)/Rab33B Q92L complex was determined at 3.47 Å resolution. Two Rab33B molecules bind to the parallel Atg16L1 coiled-coil that is located in the center of the complex, similar to other effector complexes of Rab GTPases with symmetric coiled-coils including Rab6/GCC185^38^ and Rab11/FIP2^39,40^.

The Atg16L1 model contains residues 159–208, the C-terminal end of the coiled-coil domain, which until now has not been structurally characterized. The Atg16L1 helices start diverging from residue 189 onwards due the large side chain of W194 and W194′ facing each other at the coiled-coil core and repulsive electrostatic interactions between E197 and E197′ and E201 and E201′ from the two α-helices. The Atg16L1 coiled-coil domain is highly conserved among vertebrates^41^ including these three residues, therefore these structural features likely also occur in other Atg16L1 homologues.

Rab33B interacts with the C-terminal region of Atg16L1 from E186 up to N206, which explains our observation that truncations prior to residue 210 abolished Rab33B/Atg16L1 complex formation. The Atg16L1 construct (163–210) interacts with Rab33B but forms a less stable complex than Atg16L1(153–210). The ten additional N-terminal residues likely stabilize the coiled-coil and therefore promote complex stability of the longer Atg16L1(153–210) construct with Rab33B. We prepared three Atg16L1 point mutants of residues at the complex interface: K198A, A202W and N206K. Pull-down assays showed that these Atg16L1 mutations abolished Rab33B/Atg16L1 complex formation and strongly reduced cellular co-localization of the two proteins. Mutating the aromatic triad residues F70 and W87 of Rab33B, which are important for Rab-effector interactions also interfered with Rab33B/Atg16L1 complex formation. Hence, Atg16L1 behaves like a canonical small GTPase effector protein, which binds the canonical Rab effector domain^34,42,43^.

While active Rab33B Q92L associates with the Golgi compartment (as shown in both Fig. 6b and S4a), the respective active Rab33B Q92L mutants F70A, F70E and W87A show reduced or no localization with the Golgi, suggesting that this localization might require Atg16L1. In fact, recent studies revealed that Atg16L1 shows an intrinsic ability to bind lipids^44^. The Rab33B binding site of Atg16L1 (191–208) (Fig. 1) immediately precedes the WIPI2b binding site of Atg16L1 (207–242)^23^ Our cellular localization studies show that Atg16L1, Rab33B and WIPI2b co-localize in cytoplasmic punctate structures suggesting that Atg16L1 might interact simultaneously with Rab33B and WIPI2b . However, further work is needed to understand the mechanism how Atg16L1 comes together with Rab33B and WIPI2B at the same time. The Atg16L1 mutations K198A, A202W and N206K that abolished binding to Rab33B did not affect their co-localization with WIPI2b. Atg16L2 is an isoform of Atg16L1 and both share the same domain structure but differ in their middle region (Figure S5), which we identified as responsible for interacting with Rab33B. Atg16L2 forms a complex with Atg5-Atg12 that is not recruited to the isolation membrane^16^ and Atg16L2 does not bind WIPI2b^23^. Atg16L2 also does not interact with Rab33B^16^. This can be explained by the lack of conservation of the Rab33B binding site between Atg16L1 and Atg16L2 (Figure S5). It was previously proposed that Rab33B/Atg16L1 complex formation might facilitate the recruitment of vesicles originating from the Golgi to the growing isolation membrane^30^. Thus, WIPI2b recruits the Atg12-Atg5/Atg16 complex to the site of autophagosome formation and the neighboring Rab33B and WIPI2b binding sites in Atg16L1 would bring Rab33B containing vesicles in close proximity to the isolation membrane, providing a potential source of lipids during autophagosome biogenesis.

## Methods

### Protein expression and purification

Truncated mRab33B constructs were amplified from cDNA clones (GenBank, BC065076) and cloned into the multiple cloning site 1 (MCS1) of the pETDuet-1 vector using the BamHI and NotI restriction sites. Truncated mAtg16L1 constructs were amplified from cDNA (GenBank, BC049122) and cloned into MCS2 of the pETDuet-1 vector using the NdeI and KpnI restriction sites. Mutations were introduced by the QuikChange Lightning Multi Site-Directed Mutagenesis Kit according to the manufacturer’s instructions.

pETDuet-1 mRab33B-mAtg16L1 constructs were transformed in *E. coli* BL21 competent cells. N-terminal His_6_-tagged mRab33B and untagged mAtg16L1 were co-expressed overnight at 25 °C in ZYM-5052 auto-inducing medium^45^. Cells were harvested and resuspended in 50 mM HEPES pH 7.5, 250 mM NaCl, 30 mM imidazole, 5 mM MgCl_2_, 1 mM TCEP and stored frozen at − 20 °C. Cell pellets were thawed and supplemented with one protease inhibitor tablet (cOmplete, EDTA-free Protease Inhibitor Cocktail, Roche), DNaseI, lysozyme and 1 mM MgCl_2_. Cell suspensions were stirred for 15 min at room temperature and then homogenized with a homogenizer and lysed with three repetitions in a microfluidizer M-110L (Microfluidics International Corporation). Cell debris was pelleted at 30,600 × *g* at 4 °C for 45 min. The supernatant was applied to a 5 mL HisTrap column (GE Healthcare) equilibrated with buffer A (50 mM HEPES pH 7.5, 250 mM NaCl, 30 mM imidazole, 5 mM MgCl_2_, 1 mM TCEP) using an Äkta Prime FPLC system at 4 °C. The column was washed with 13 column volumes (CV) buffer A. His-tagged protein complexes were eluted with a gradient over 12 CV to 100% buffer B (50 mM HEPES pH 7.5, 250 mM NaCl, 400 mM imidazole, 5 mM MgCl_2_, 1 mM TCEP). The eluted complex was concentrated and then applied to a Superdex 200 16/60 HiLoad column (GE Healthcare) and run with gel filtration buffer (30 mM HEPES pH 7.5, 150 mM NaCl, 2 mM MgCl_2_, and 1 mM TCEP). Fractions were pooled and concentrated to 30–45 mg/mL. Purified proteins were aliquoted, flash cooled with liquid nitrogen and stored at − 80 °C.

### Ni-sepharose pull-down experiments

For Ni-Sepharose pull-down experiments^46,47^. pETDuet-1 mRab33B–mAtg16L1 constructs were expressed in *E. coli* Rosetta 2(DE3) cells. Lysed cell debris was pelleted at 30,600 × *g* at 4 °C for 45 min. 10 mL supernatant was incubated with 1 mL Ni-Sepharose beads for 1 h under constant rotation at 4 °C. Beads were centrifuged at 1,620 × *g* for 5 min at 4 °C and the flow through was discarded. Beads were washed three times with 5 mL buffer A. Proteins were then eluted twice with 1.5 mL buffer B. Elution fractions were pooled and analyzed by Schägger gel electrophoresis followed by western blotting. Membranes were blocked in 3% BSA and probed with Penta His HRP conjugate antibody (1:1,500) from Qiagen GmbH to detect His-tagged Rab33B or blocked in 5% skim milk and probed with rabbit anti-Atg16L primary antibody (1:2,000) from MBL Life Science and goat anti-rabbit IgG (HRP-labeled) (1:1,000) secondary antibody from BioRad Laboratories to detect Atg16L1. Signals were detected using Western Lightening Plus-ECL solution from PerkinElmer with an Imageready LAS-1000 CCD camera (Fujifilm).

### Crystallization and structure determination

For crystallization, Rab33B(30–202) Q92L-mAtg16L1(153–202) was purified by size exclusion chromatography using 20 mM HEPES pH 7.5, 150 mM NaCl, 2 mM MgCl_2_, 1 mM TCEP hydrochloride, 10 µM GTPγS as a running buffer. Needle-like crystals were grown in hanging drops in 24-well Linbro plates using 3 µL of protein complex (48 mg/ml), 2 µl of 0.1 M MES monohydrate pH 6.5, 0.2 M sodium chloride, 10% (w/v) PEG 4,000 and 0.5 µl of 1 M spermine at 20 °C. Crystals were soaked in crystallization solution supplemented with 25% ethylene glycol before flash cooling in liquid nitrogen. Data were collected at 100 K at beamline X06SA (Swiss Light Source, Paul Scherrer Institute, Villigen, Switzerland).

A native dataset with a 360° oscillation range was collected at 1 Å wavelength (Table 1). Data were processed with the XDS software package^48^. The structure was determined by molecular replacement using Phaser_MR within the PHENIX program suite^49^ with GppNHp-bound Rab33 (PDB accession code: 1Z06^34^ as a search model. Three additional helical regions were visible in the calculated electron density map. The complex structure was then determined by a second round of molecular replacement using the *S. cerevisiae* Atg16 coiled-coil domain (PDB code: 3A7O^12^ as a search model. The asymmetric unit contained six Rab33B molecules and three Atg16L1 dimers. Manual model building was done with COOT^50^ and refinement was done with PHENIX^49^. Non-crystallographic symmetry (NCS) restraints and grouped B-factors have been used to reduce the number of independently refined parameters in refinement. Residues revealing strong conformational differences between multiple molecules present in the asymmetric unit have been excluded from NCS restraints for final cycles of refinement. The atomic model has been verified by a calculation of composite omit map as implemented in PHENIX. Figures were prepared with PyMOL^51^.

**Table 1:** X-ray data collection and refinement statistics.

**Data collection**	
Space group	P21
**Cell dimensions**	
a, b, c (Å) α, β, γ (°)	48.4, 204.9, 107.2 90.0, 92.6, 90.0
Resolution range (Å)	48.4—3.47 (3.6—3.47)
Total reflections Unique reflections Multiplicity Completeness (%) Mean I/ σ Wilson B factor (Å^2^) R_meas_ (%) CC_1/2_	90,400 (6,535) 25,983 (2,227) 3.5 96.3 (79.9) 7.9 (2.2) 66.4 22.9 (89.2) 98.3 (72.4)
**Refinement**	
R_work_ R_free_	0.206 (0.215) 0.239 (0.246)
Molecules/AU Number of protein residues included in model:	12 A: 31–202 B: 31–183, 187–202 C: 31–202 D: 31–136, 140–181, 185–202 E: 30–202 F: 30–202 I: 159–208 J: 160–208 K: 159–208 L: 159–208 M: 160–208 N: 160–208
Number of non-hydrogen atoms Macromolecules Ligands	10,590 10,392 198
B-factors (Å^2^) Macromolecules Ligands	57.0 57.3 41.1
**Structure validation**	
Ramachandran favored (%) Ramachandran allowed (%) Ramachandran outliers (%) Rotamer outliers (%) Clashscore	97 3 0 0.46 6
**RMSD deviations**	
Bond lengths (Å) Bond angles (°)	0.005 0.70

Values in parentheses refer to the highest resolution shell, Ramachandran statistics were calculated with Molprobity.

### Cell lines, transient transfection, co-immunoprecipitation and immunostaining

HEK293T, Cos-7 and HeLa S6 cells were cultivated using a method described in^52^. HEK293T cells were used to perform co-immunoprecipitation (CoIP) assay, optimizing the the method reported in^53^. Precisely, cells were seeded one day before transfection using Lipofectamin 2000 (Invitrogen) according to the manufacturer's instructions. After 24 h, transfected cells were lysed in 600 µL buffer containing 50 mM HEPES pH 7.4, 150 mM NaCl, 1 mM MgCl_2_, 1% TX-100 for 15 min at 4 °C. The lysate was centrifuged at 10,000 × *g* for 10 min. 30 µL of supernatant was used as input. The rest of the sample was used for the co-immunoprecipitation assay.

The pre-cleared supernatant was incubated with 10 µg of specific antibodies (mouse anti-V5 (1:2,000) from Santa Cruz Biotechnology or rabbit anti-GFP from Synaptic Systems) for 2 h under constant rotation. Next, the supernatant was transferred to pre-washed beads (30 µL of dynabeads protein A, Thermo Fisher Scientific) and incubated for an additional hour under constant rotation. Each step was carried out at 4 °C or on ice. Subsequently, the beads were washed three times with 1 mL lysis buffer, and in the fourth wash the beads were transferred to a new tube, where they were subjected to two additional washes. Finally, proteins were eluted from the beads using 4 × LDS Sample Buffer (NuPAGE, Invitrogen) in the presence of 10% beta-mercaptoethanol and boiled for 10 min at 70 °C. 5 µL of the input and 10 µL of the IP sample were loaded onto a SDS-PAGE gel and then subjected to Western blotting. The membranes were probed with rabbit anti-GFP (1:10,000) from Synaptic Systems or mouse anti-V5 (1:2000) from Santa Cruz Biotechnology; LC3B (1:1,000) from nanoTools Antibodies; or WIPI2 (1:500) from Merck.

Immunostaining was done as described before^47,52^. Briefly, transiently transfected HeLa and Cos-7 cells were washed once with PBS to remove the serum. The cells were fixed using 4% paraformaldehyde for 15 min at RT. The fixative was removed, and the cells were washed 3 times 5 min each with PBS. Afterwards the cells were blocked with 10% normal goat serum and 0.2% Triton-X100 in PBS for 1 h. The coverslips were inverted on top of a drop of 45–50 μL of specific antibodies (rabbit anti-V5 primary antibody (1:500) from GeneTex, mouse anti-WIPI2 (1:200) from Merck), and mouse Golgin-97 (1:200) from Thermo,) diluted in blocking buffer. The incubation was performed in a dark and humidified chamber for 1 to 2 h at RT or o/n at 4 °C. The coverslips were washed 3 times for 5 min each with PBS and incubated again following the same procedure with fluorescently labelled secondary antibodies (mouse and/or anti-rabbit Cy3 and Cy5 (1:600) from Dianova GmbH) for 1 h at RT. Finally, the coverslips were mounted on microscope slides using VECTASHIELD HardSet Mounting Medium with DAPI (Vector Laboratories). Images were acquired using an epifluorescence microscope (Axioverter 200 M, ZEISS for the COS cells); the confocal SP2 LEICA microscope with 63X oil immersion objective or high resolution microscope SIM (Structural illumination microscope) using Plan-Apochromat 63x/1.4 Oil DIC M27 for the HeLa cells. JACoP plugin was used for the colocalization studies and imageJ to quantify western blots.

## Supplementary information

Supplementary file1

## Data Availability

Coordinates and structure factors have been deposited in the Protein Data Bank under accession code 6SUR. Other data are available from the corresponding authors upon reasonable request.
