# Supplementary material for: Crystal structure of the Rab33B/Atg16L1 effector complex
Source: Sci Rep. 2020 Jul 31;10:12956. doi: 10.1038/s41598-020-69637-0 (PMC7395093; doi:10.1038/s41598-020-69637-0)
Supplement: Supplementary file 1 — Supplementary file1 [file 41598_2020_69637_MOESM1_ESM.docx]

**Supplementary Information**

**Crystal structure of the Rab33B/Atg16L1 effector complex**

Janina Metje-Sprink^1,3*^, Johannes Groffmann^1^, Piotr Neumann^2^, Brigitte Barg-Kues^1^, Ralf Ficner^2^, Karin Kühnel^1,4*^, Amanda M. Schalk^1,5^ and Beyenech Binotti^1,6*^

^1^Department of Neurobiology, Max-Planck-Institute for Biophysical Chemistry, 37077 Göttingen, Germany

^2^ Department of Molecular Structural Biology, Institute of Microbiology and Genetics, GZMB, Georg-August-University Göttingen, 37077 Göttingen, Germany

^3^present address: Institute for Biosafety in Plant Biotechnology, Julius Kuehn-Institute, 06484 Quedlinburg, Germany

^4^ present address: Nature Communications,4 Crinan Street, N1 9XW, London, United Kingdom

^5^ present address: Department of Biochemistry and Molecular Genetics, University of Illinois at Chicago, Chicago, Illinois 60607, USA

^6^ present address: Department of Biochemistry University of Würzburg, 97074 Würzburg, Germany

*corresponding authors: J.M.-S., K.K., B.B.
Janina Metje-Sprink, Email: janina.metje@julius-kuehn.de
Karin Kühnel, Email: karink1303@gmail.com>
Beyenech, Binotti, Email: beyenech.binotti@uni-wuerzburg.de


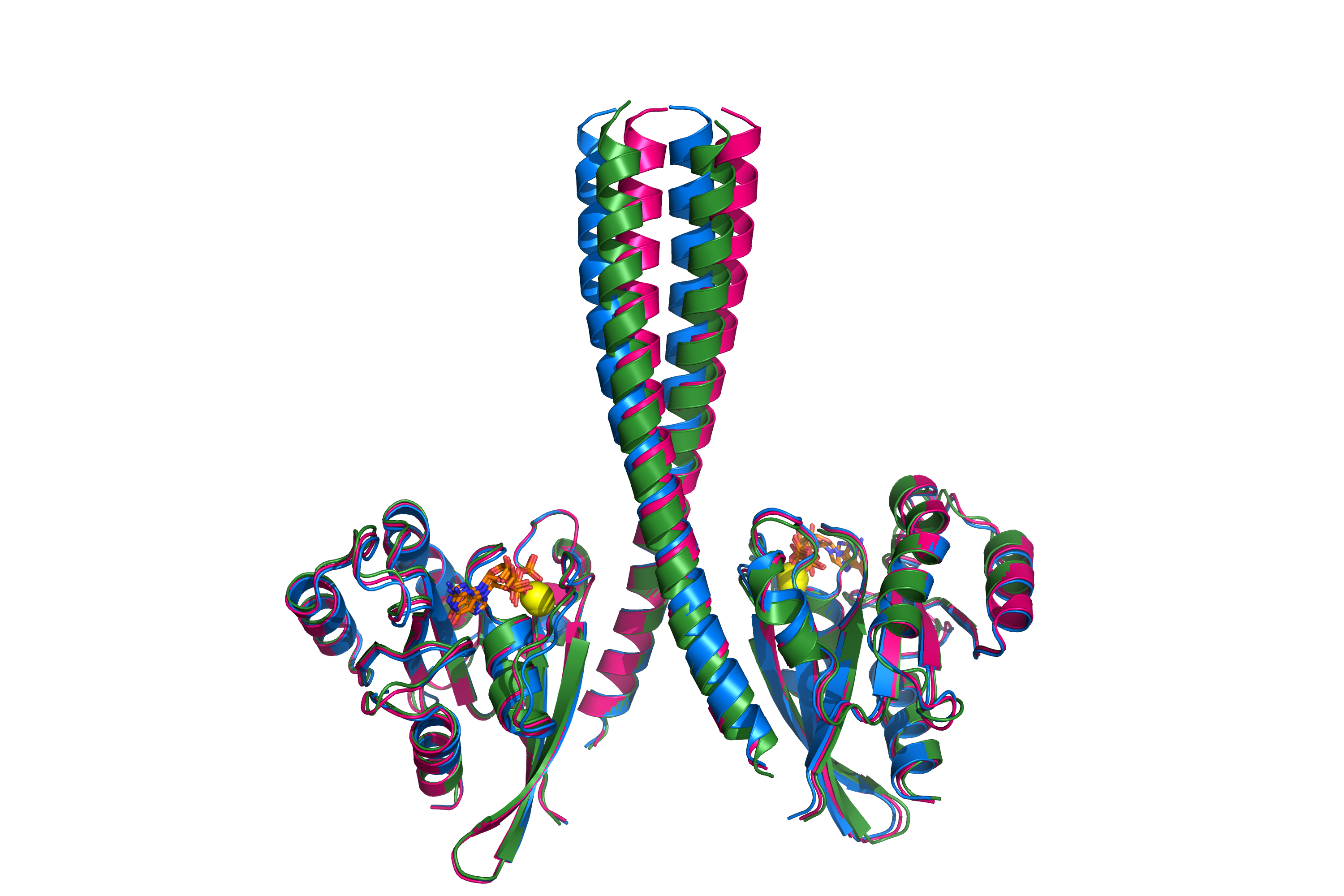


Figure S1. Superimposition of the three Rab33B(30-202)Q92L-Atg16L1(153-210) complexes in the asymmetric unit.


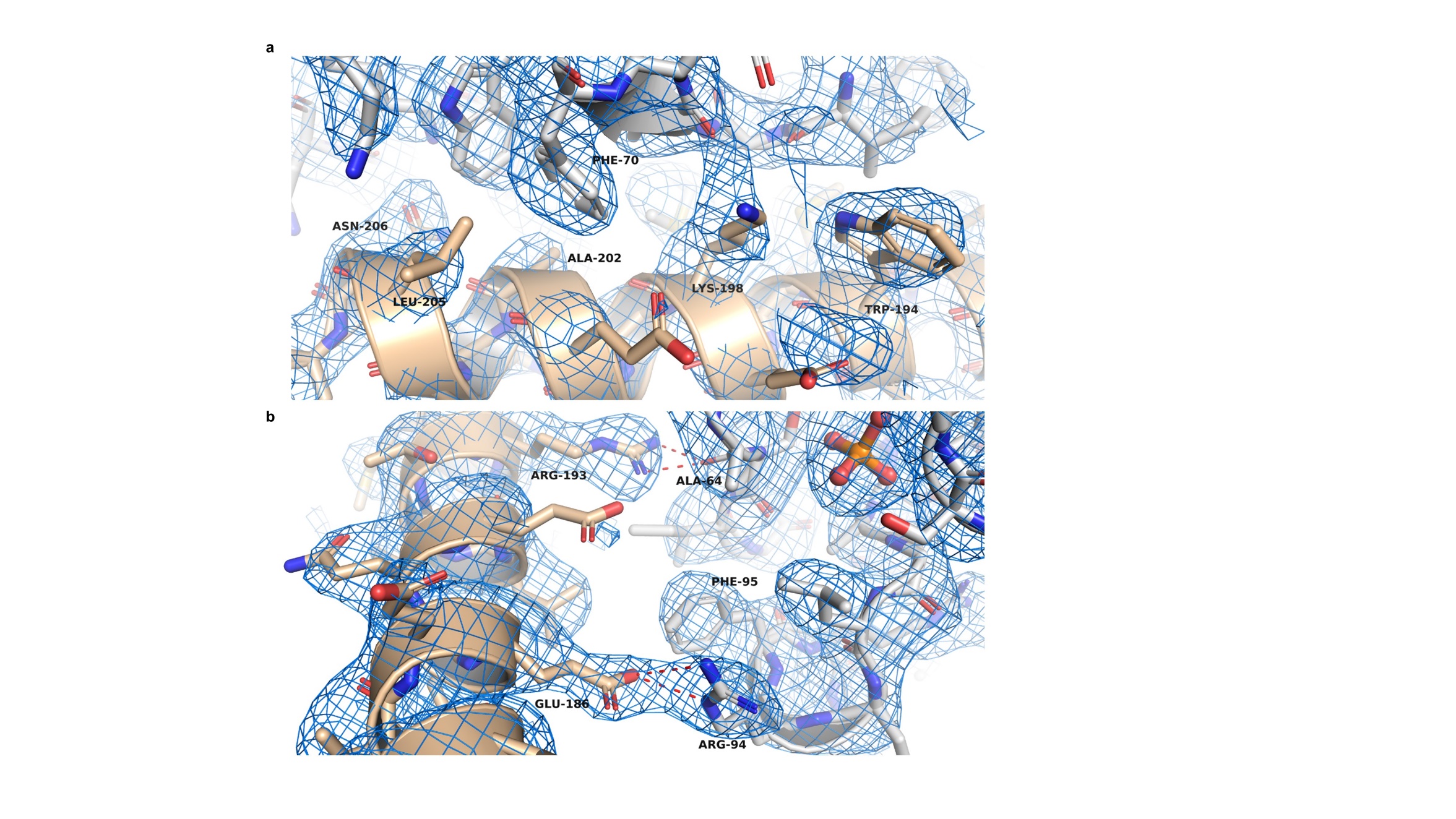


Figure S2. **Composite omit electron density map of Rab33-Atg16 interaction sides**.
a: Rab33-Atg16 interaction between residue Rab33 F70 and Atg16 K198, A202 and N206. b: Salt bridge formation between Rabb33 and Atg16. The Rab33B molecule is colored in light brown and mAtg16L1 is shown in grey. Rab33 and Atg16 residues involved in complex formation drawn in stick representation. Salt bridges are shown as red dashed lines. The composite omit 2mFo-DFc map contoured at 1σ is shown in blue.


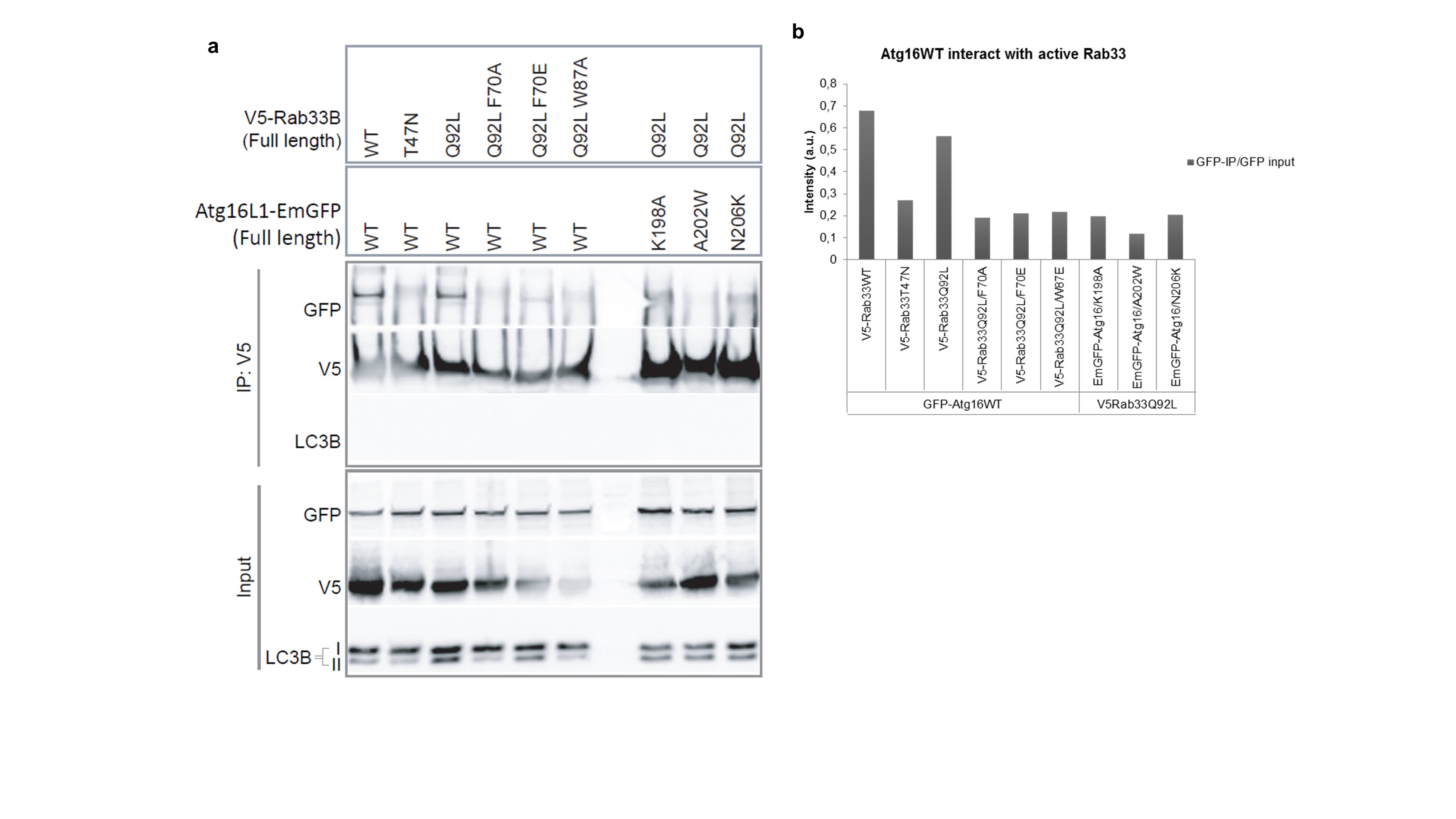


Figure S3. **V5** co-immunoprecipitation of Atg16L1-EmGFP and V5-Rab33B variants.

**a:** Overexpression was done in HEK293 cells. Western blots were probed with either anti-GFP, anti-V5 or anti-LC3B antibodies. IP: Immunoprecipitation, IB: immunoblot. **b:** Band intensities were calculated normalizing the EmGFP-Atg16 (GFP-IP) western blot band intensities against GFP input intensity. Uncropped blots are shown in Figure S9.


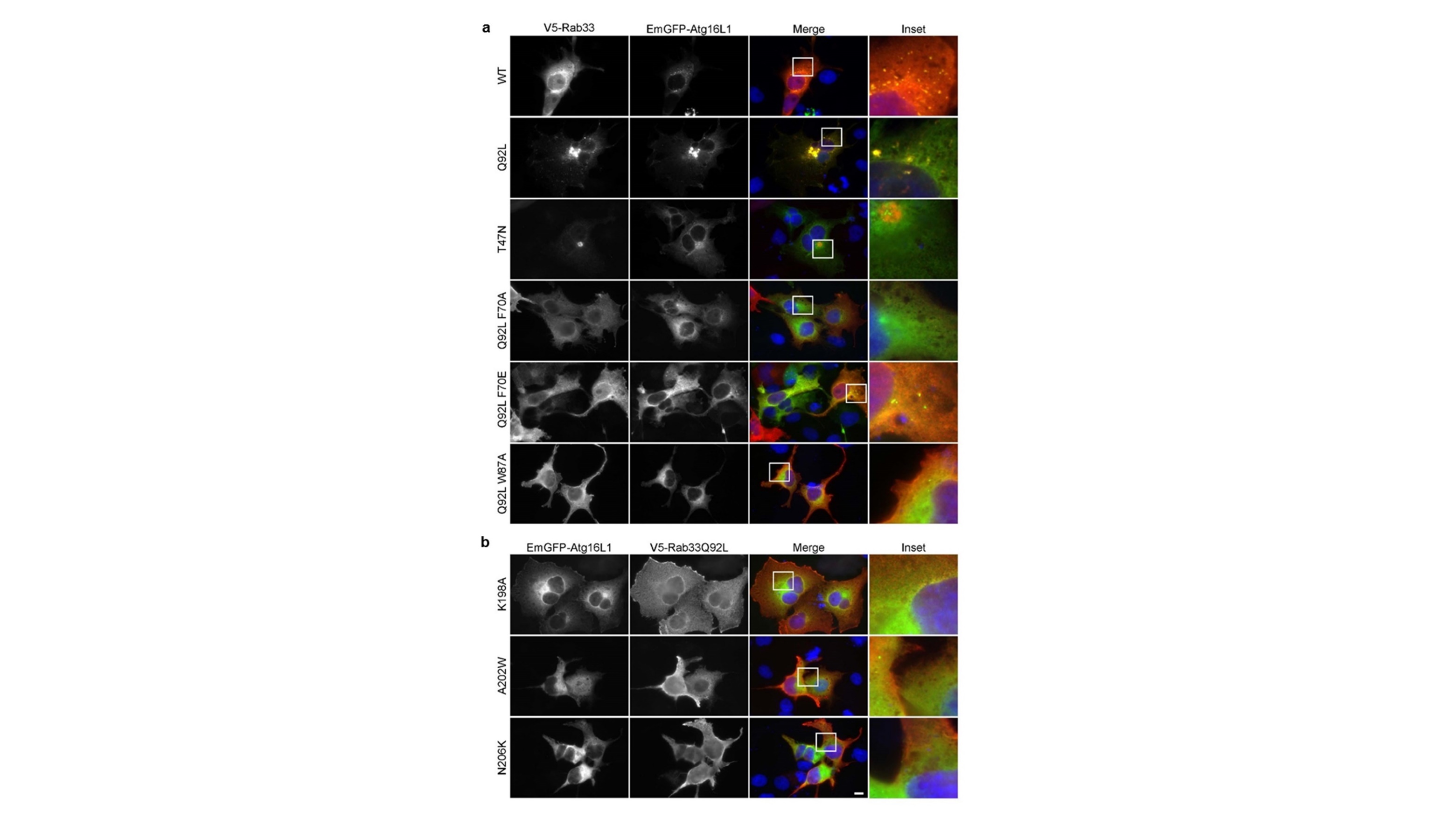


Figure S4. Intracellular distribution of EmGFP-Atg16L1 and V5-Rab33B WT/ Q92L or T47N.

EmGFP-Atg16L1 and V5-Rab33B variants were transiently expressed in Cos-7 cells. Coexpression of **a**: EmGFP-Atg16L1 WT and V5-Rab33B WT or mutants, **b**: EmGFP-Atg16L1 mutants and V5-Rab33B Q92L. While all Rab33B mutants do not show any overlap with Atg16WT (**a**); Atg16L1K198A causes relocalization of Rab33Q92L to the cell surface (**b,** row 1). Scale bar, 10 µm. Figures are representative for three biological replicates.


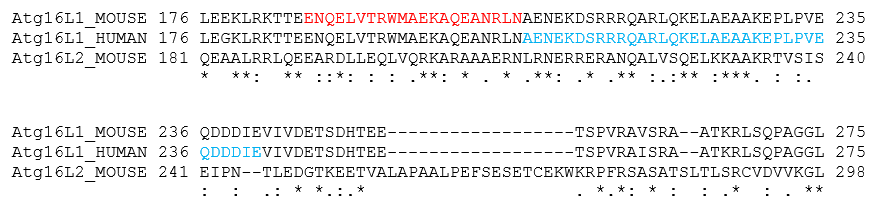


Figure S5. **Sequence alignment of human Atg16L1 (Q676U5), murine Atg16L1 (Q8C0J2-3) and murine Atg16L2 (Q6KAU8-1).**

The Rab33B binding site is colored red and the WIPI2b binding site is shown in blue. Both sites are conserved in human and murine Atg16L1 but not in Atg16L2. Sequence alignment was done with Clustal Omega [^47^](#_ENREF_47).

Figure S6. **Uncropped images of blots shown in Figure 1b**

**
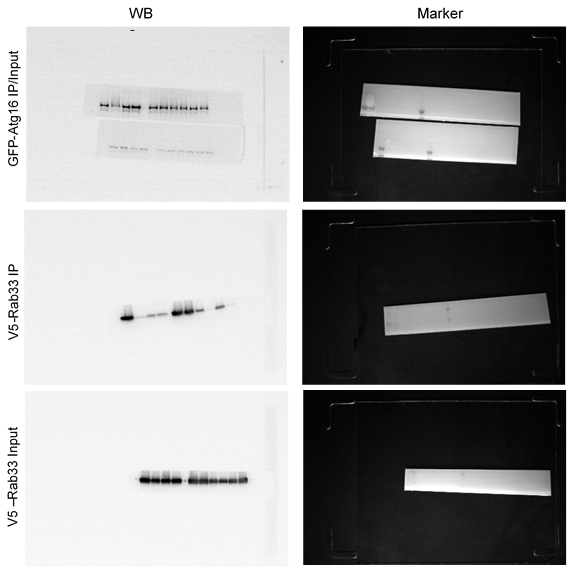
**


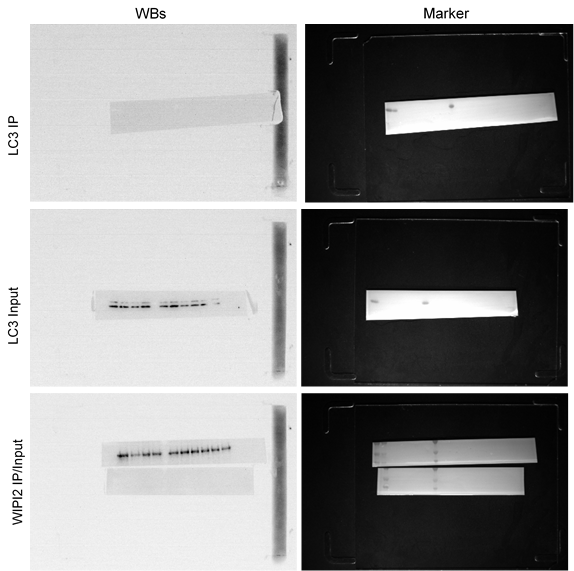


Figure S7. **Uncropped images of blots shown in Figure 5a**

Figure S8. **Uncropped images of blots shown in Figure 5c**


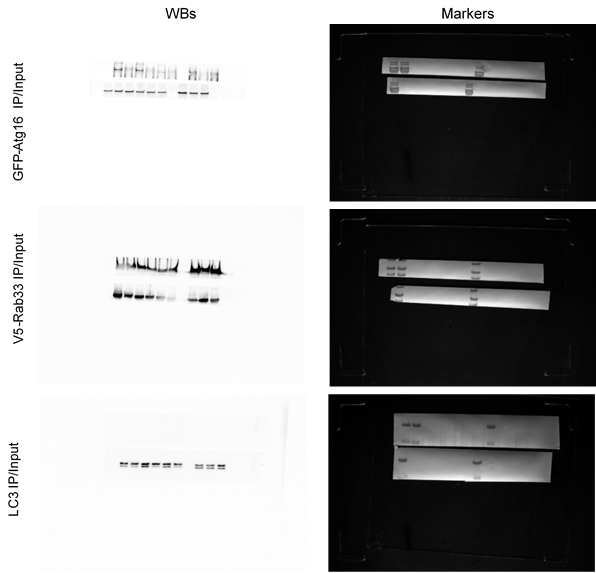


Figure S9. **Uncropped images of blots shown in Figure S3**
